# Antimicrobial Drug Resistance in Pathogens Causing Nosocomial Infections at a University Hospital in Taiwan, 1981-1999

**DOI:** 10.3201/eid0801.000454

**Published:** 2002-01

**Authors:** Po-Ren Hsueh, Mei-Ling Chen, Chun-Chuan Sun, Wen-Hwei Chen, Hui-Ju Pan, Li-Seh Yang, Shan-Chwen Chang, Shen-Wu Ho, Chin-Yu Lee, Wei-Chuan Hsieh, Kwen-Tay Luh

**Affiliations:** * National Taiwan University College of Medicine, Taipei, Taiwan; † National Taiwan University Hospital, Taipei, Taiwan

**Keywords:** nosocomial infections, antimicrobial resistance, Taiwan, bacterial pathogens, hospital-based surveillance

## Abstract

To determine the distribution and antimicrobial drug resistance in bacterial pathogens causing nosocomial infections, surveillance data on nosocomial infections documented from 1981 to 1999 at National Taiwan University Hospital were analyzed. During this period, 35,580 bacterial pathogens causing nosocomial infections were identified. *Candida* species increased considerably, ranking first by 1999 in the incidence of pathogens causing all nosocomial infections, followed by *Staphylococcus aureus* and *Pseudomonas aeruginosa*. *Candida* species also increased in importance as bloodstream infection isolates, from 1.0% in 1981-1986 to 16.2% in 1999. The most frequent isolates from urinary tract infections were *Candida* species (23.6%), followed by *Escherichia coli* (18.6%) and *P. aeruginosa* (11.0%). *P. aeruginosa* remained the most frequent isolates for respiratory tract and surgical site infections in the past 13 years. A remarkable increase in incidence was found in methicillin-resistant *S. aureus* (from 4.3% in 1981-1986 to 58.9% in 1993-1998), cefotaxime-resistant *E. coli* (from 0% in 1981-1986 to 6.1% in 1993-1998), and cefotaxime-resistant *Klebsiella pneumoniae* (from 4.0% in 1981-1986 to 25.8% in 1993-1998). Etiologic shifts in nosocomial infections and an upsurge of antimicrobial resistance among these pathogens, particularly those isolated from intensive care units, are impressive and alarming.

The emergence of resistance to antimicrobial agents is a global public health problem, particularly in pathogens causing nosocomial infections *1*–*5*. Antimicrobial resistance results in increased illness, deaths, and health-care costs *1*,*2*,*6*–*10*. The distribution of pathogens causing nosocomial infections, especially antimicrobial-resistant pathogens, changes with time and varies among hospitals and among different locations in the same hospital *11*–*15*. The increasing number of immunocompromised patients and increased use of indwelling devices, as well as widespread use of antimicrobial agents in hospital settings, particularly in intensive care units (ICUs), contributes to antimicrobial resistance among pathogens causing nosocomial infections *1*,*4*,*6*,*10*.

Surveillance data reported by the National Nosocomial Infections Surveillance (NNIS) System for 1993-1997 compared with January-November 1998 show a continuing increase in antimicrobial-resistant pathogens associated with nosocomial infections in ICU patients from U.S. hospitals *2*. The increase is particularly marked for vancomycin-resistant enterococci (VRE) (55%), methicillin-resistant *Staphylococcus aureus* (MRSA) (31%), third-generation cephalosporin-resistant *Escherichia coli* (29%), imipenem-resistant *Pseudomonas aeruginosa* (32%), and quinolone-resistant *P. aeruginosa* (89%) *2*. Studies since 1990 have clearly demonstrated that *Candida* species continue to be an important cause of nosocomial infections, particularly of bloodstream infections (BSI). Furthermore, the proportion of BSI caused by *Candida* species other than *C. albicans* is increasing *14*,*16*–*20*.

We describe the distribution of major bacterial pathogens causing nosocomial infections from 1981 to 1999 in National Taiwan University Hospital and demonstrate the emergence of antimicrobial drug resistance among these pathogens during this period.

## Materials and Methods

### Data Collection

National Taiwan University Hospital is a 2,000-bed tertiary referral center in Taipei, northern Taiwan. Available data for inpatient-days at the hospital ranged from 294,946 in 1990 to 566,165 in 1999. The number of ICU beds increased from 40-50 before 1993 to 100-120 in 1998-99. The Nosocomial Infection Control Committee of the hospital was established in 1980 to identify pathogens causing nosocomial infections and to obtain and analyze antimicrobial susceptibility results of these pathogens from the hospital’s clinical microbiology laboratory. NNIS definitions were used for nosocomial infections (e.g., bloodstream; respiratory tract, including lower respiratory tract and pneumonia; urinary tract; and surgical site infections) *21*,*22*. Isolates were considered nosocomial if the culture was dated >2 days after admission. All isolates were identified by standard methods and confirmed by using Vitek or API products (bioMerieux Vitek, Inc., Hazelwood, MO). For determining the percentage of resistance, the same organisms from multiple blood cultures or from the same sources with identical antibiotype were considered a single isolate. The amount of use for each indicated antimicrobial agent (including oral and parenteral forms) was expressed in grams per 1,000 inpatient-days.

### Antimicrobial Susceptibility Testing

Antimicrobial susceptibility testing of the bacterial isolates was performed by the disk diffusion method as described by the National Committee for Clinical Laboratory Standards (NCCLS) *23*. *S. aureus* ATCC 25923, *E. coli* ATCC 25922, and *P. aeruginosa* ATCC 27853 were included as control strains. Interpretive criteria for susceptibility or resistance followed NCCLS guidelines *23*. For this report, we present susceptibility data for penicillin, oxacillin, vancomycin, gentamicin, cefotaxime, ceftazidime, imipenem, and ciprofloxacin. The susceptibility data for imipenem and ciprofloxacin were available only since 1993; those for other agents were available from 1981 to 1999.

Antifungal susceptibility testing of amphotericin B and fluconazole against 150 blood isolates of *Candida* species collected from October 1997 to September 1999 was performed by the E test (AB BIODISK, Solna, Sweden) according to the manufacturer’s instructions. Quality control was performed by testing *C. parapsilosis* ATCC 2019 and *C. krusei* ATCC 6258. MIC results were interpreted in accordance with NCCLS guidelines (*24*).

## Results

**Figure 1:**
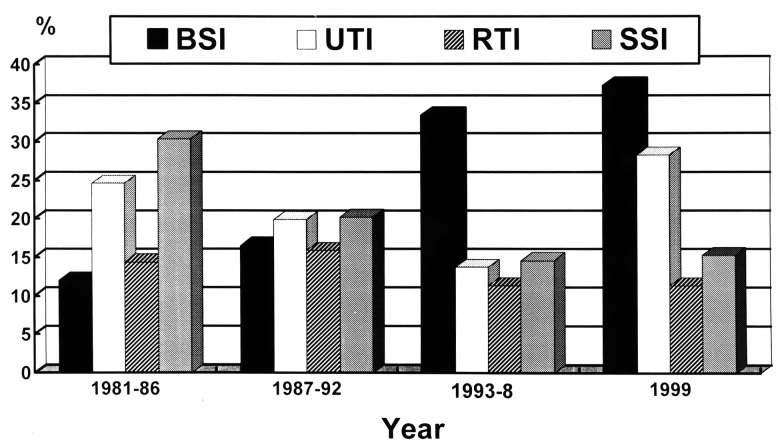
Rates of four major nosocomial infections expressed as number of infections per 10,000 patient-days at National Taiwan University Hospital from 1991 to 1999. BSI, bloodstream infection; UTI, urinary tract infection; SSI, surgical site infection; RTI, respiratory tract infection.

During the 19-year period, 35,580 bacterial pathogens causing nosocomial infections were identified. The hospital’s overall rate of nosocomial infections during the 19-year period ranged from 3.9% to 6.1%. For the four major sites of nosocomial infections, data are presented as numbers of infection per 10,000 patient-days from 1991 to 1999 (Figure 1). BSI ranked first in nosocomial infection sites in 1999, followed by urinary tract, surgical site, and respiratory tract infections.

Data for the catheter- and ventilator-days of the hospitalized patients were not available. However, the mean percentages of patients who stayed in ICUs (six medical ICUs and six surgical ICUs) and used urinary catheters, arterial and central venous catheters, and ventilators were 75.0%, 77.9%, 63.2%, respectively, in 1996 and 79.4%, 81.4%, and 65.2%, respectively, in 1999. The incidence (number of infections/patient-days x 1,000) of urinary catheter-related urinary tract infection, vascular catheter-associated BSI, and ventilator-associated pneumonia in the ICUs was 4.4, 5.6, and 2.7, respectively, in 1996 and 6.0, 7.1, and 3.4, respectively, in 1999.

Gram-negative bacteria remained the predominant pathogens (66.1% in 1981, 51.3% in 1993, and 53.4% in 1999); however, incidences of fungal infections have increased recently (Table 1). In a comparison of data grouped into four time periods (1981-1986, 1987-1992, 1993-1998, and 1999), *E. coli* decreased from 12.1% of all nosocomial infections in 1981-1986 to 9.5% in 1993-1998; however, infections caused by *S. aureus* increased from 5.2% (1981-1986) to 12.1% (1993-1998). *Candida* species showed a considerable increase (from 3.7% in 1981-1986 to 16.2% in 1999) and have ranked first since 1993, followed by *S. aureus* and *P. aeruginosa* (Table 1).

**Table 1:** Incidences of 10 top-ranking pathogens causing nosocomial infections and infections from four body sites at National Taiwan University Hospital, 1981 to 1999

Pathogen	% of indicated pathogen causing nosocomial infection (1981-1986/1987-1992/1993-1998/1999)^a^				
	All	BSI	RTI	UTI	SSI
*Candida* spp.	3.7/9.1/14.4/16.2	1.0/9.2/16.4/16.2	2.0/5.8/2.1/2.2	8.4/16.0/23.6/14.3	2.4/5.1/5.9/6.4
*Staphylococcus aureus*	5.2/9.1/12.1/12.0	5.2/9.3/11.5/13.0	4.0/8.4/16.9/12.6	1.4/2.6/3.3/2.1	5.5/5.2/13.0/15.4
*Pseudomonas aeruginosa*	12.7/14.0/11.1/11.8	10.0/9.4/7.2/7.8	19.6/21.9/23.8/25.7	11.7/11.2/11.0/10.4	11.1/17.4/14.3/16.0
*Escherichia coli*	12.1/8.4/9.5/9.9	18.7/9.7/8.7/9.0	4.8/2.4/3.5/3.7	19.1/19.9/18.6/18.4	11.7/5.8/5.8/6.8
*Klebsiella pneumoniae*	8.1/5.5/7.2/6.8	11.6/6.6/7.7/7.0	10.9/9.4/11.5/10.8	9.0/7.0/8.6/8.2	6.9/3.5/4.2/4.6
*Enterobacter* spp.	6.0/7.6/7.4/6.4	8.0/8.6/7.3/6.9	5.2/8.1/11.8/8.6	9.0/8.4/6.7/6.3	4.5/7.9/7.8/5.5
*Enterococcus* spp.	8.8/7.8/6.7/6.2	8.7/6.2/6.3/7.6		11.6/9.7/8.1/6.5	10.1/12.6/9.6/7.9
*Acinetobacter* spp.	4.4/5.1/4.9/5.4	6.1/8.8/7.2/7.6	11.0/13.4/9.3/13.0		
CoNS	2.8/6.9/6.6/5.1	2.7/8.5/7.9/4.9			3.2/7.1/9.1/6.8
Other NFGNB	5.7/6.1/4.8/4.1	5.9/7.7/6.8/6.7	12.3/13.1/7.8/8.2	7.4/6.5/4.4/2.8	
*S. marcescens*			3.5/1.9/3.7/4.1		
*Proteus* spp.			3.2/2.2/2.4/1.1	3.8/3.8/3.8/3.7	
*Citrobacter* spp.				5.9/4.4/2.4/2.4	
*Viribans streptococci*					5.2/5.8/4.2/3.5
*Bacteroides* spp.					9.7/4.7/5.0/3.1

^a^Abbreviations: BSI = bloodstream infection; RTI = respiratory tract infection; UTI = urinary tract infection; SSI = surgical site infection; CoNS = coagulase-negative staphylococci; NFGNB = nonfermentative gram-negative bacilli.

*Candida* species, *S. aureus,* and *Acinetobacter* species were also important bloodstream isolates (Table 1), increasing from 1.0%, 5.2%, and 6.1%, respectively, in 1981-1986 to 16.2%, 13.0%, and 7.6%, respectively, in 1999. Although *E. coli* was also a frequent isolate, it declined in percentage of all BSI from 18.7% in 1981-1986 to 9.0% in 1999. Other pathogens declining in percentage of BSI from 1981-1986 to 1999 included *K. pneumoniae,* *P. aeruginosa*, and *Enterococcus* species.

From 1992 to 1999, 1,065 isolates of *Candida* species were recovered from patients with nosocomial BSIs. *C. glabrata* (8.8%) ranked fourth in incidence behind *C. albicans* (59.3%), *C. tropicalis* (17.6%), and *C. parapsilosis* (8.2%) in 1999. Only four blood isolates of *C. krusei* were identified during the 8-year period.

The most frequent isolates from urinary tract infections in 1993-1998 were *Candida* species (23.6%), followed by *E. coli* (18.6%) and *P. aeruginosa* (11.0%). However, in 1999, *E. coli* (18.4%) replaced *Candida* species (14.3%) as the top-ranking pathogen causing urinary tract infections. *P. aeruginosa* remained the most frequent isolate for respiratory tract and surgical site infections in the past 13 years. *Candida* species increased in incidence in surgical site infections, from 1.8% in 1981-1986 to 6.4% in 1999. Among pathogens causing respiratory tract infections, *Acinetobacter* species ranked fifth in 1993-1998 (9.3%) but second in 1999 (13.0%); *Candida* species accounted for only 2.2% in 1999.

The distributions of selected antimicrobial drug-resistant pathogens causing all nosocomial infections and BSIs of patients hospitalized in intensive care units or general wards is shown in Table 2. Antimicrobial drug-resistant pathogens causing BSIs that increased markedly over the study period were methicillin-resistant *S. aureus* (4.3% in 1981-1986, 58.9% in 1993-1998, and 69.2% in 1999), cefotaxime-resistant *E. coli* (0% in 1981-1986, 6.1% in 1993-1998, and 12.5% in 1999), and cefotaxime-resistant *Klebsiella pneumoniae* (4.0% in 1981-1986, and 25.8% in 1993-1998). The frequencies of these three resistant pathogens were considerably higher in isolates from ICUs than those from general wards (84.6% vs. 48.3% for MRSA, 17.1% vs. 5.1% for cefotaxime-resistant *E. coli*, and 51.1% vs. 18.3% for cefotaxime-resistant *K. pneumoniae* in 1993-1998). The incidence of methicillin-resistant coagulase-negative staphylococci (MRCoNS) remained high (72%-90%) during the 19-year period.

**Table 2:** Selected antimicrobial resistant pathogens associated with nosocomial infection at National Taiwan University Hospital from 1981 to 1999

Resistant pathogen	% resistance (all patients/in intensive care unit patients/in patients in general wards)			
	1981-1986	1987-1992	1993-1998	1999
Methicillin-resistant *Staphylococcus aureus*	20.2/27.8/19.5	31.4/58.5/26.6	64.8/86.9/56.7	69.3/87.4/60.2
Methicillin-resistant CoNS*^a^*	72.2/88.2/70.8	74.0/83.9/71.5	79.0/88.7/75.1	90.6/90.9/90.5
Penicillin-resistant enterococci	5.9/6.8/5.8	20.7/30/19.6	19.5/22.6/18.7	35.9/40.9/27.8
Gentamicin-resistant enterococci	0.0/0.0/0.0	71.0/71.4/70.9	61.5/67.2/60.0	50.0/39.3/55.2
Cefotaxime-resistant *Escherichia coli*	1.6/0.0/1.7	2.8/8.6/2.5	6.8/13.1/6.2	12.3/10.3/12.7
Cefotaxime-resistant *Klebsiella pneumoniae*	4.9/8.8/3.9	7.4/16.9/5.6	22.8/50.5/15.6	16.5/40.0/9.3
Cefotaxime-resistant *Enterobacter *spp.	35.8/52.4/32.4	49.7/55.7/47.7	57.6/67.0/53.6	50.9/61.8/46.2
Cefotaxime-resistant *Pseudomonas aeruginosa*	24.2/20.0/25.0	16.1/24.3/12.1	10.2/16.9/7.8	11.2/17.6/8.1
Imipenem-resistant *P. aeruginosa*	NA	NA	8.5/18.2/4.9	6.7/4.5/7.7
Ciprofloxacin-resistant *P. aeruginosa*	NA	NA	8.7/11.6/5.8	14.0/18.2/2.9
Imipenem-resistant *Acinetobacter baumannii*	NA	NA	6.7/9.1/4.4	12.5/23.3/7.6

^a^CoNS = coagulase-negative staphylococci; NA = not available.

**Figure 2:**
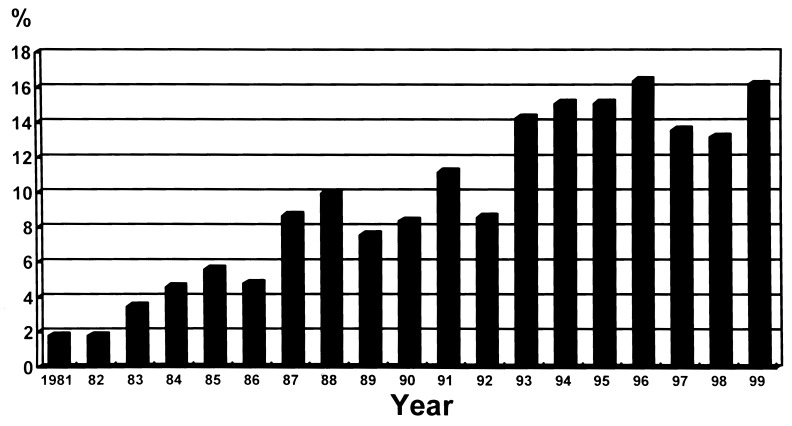
Changes in major antimicrobial-resistant nosocomial pathogens in relation to ceftazidime use at National Taiwan University Hospital from 1991 to 1999.

Ceftazidime use is associated with trends of several antimicrobial-resistant pathogens during the period 1991 to 1999 (Figure 2). Restriction of third-generation cephalosporins (particularly ceftazidime) was implemented in 1997. In 1999, resistance to cefotaxime in *K. pneumoniae* diminished; however, resistance to cefotaxime in *E. coli* and resistance to ceftazidime in *P. aeruginosa* slightly increased.

**Figure 3:**
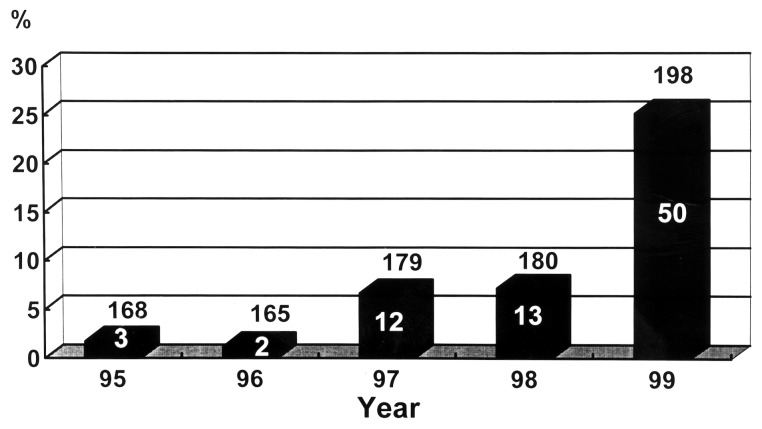
Incidences of vancomycin-resistant enterococci (VRE) among all enterococcal isolates causing nosocomial infections in relation to vancomycin use at National Taiwan University Hospital, 1995-1999. Numbers above the bars denote the number of enterococcal isolates causing nosocomial infections. Numbers within the bars denote the numbers of VRE.

The first clinical isolate of VRE was recognized in 1995 *25*,*26*. Since then, 80 isolates of VRE (49 of *E. faecalis* and 31 of *E. faecium*) have been recovered from hospitalized patients. The incidence of VRE in isolates causing nosocomial infection increased from 1.8% in 1995 to 6.7% in 1997 and 25.2% in 1999 (Figure 3). Among these VRE isolates, 4 were from blood, 15 from urine, and the rest were pus or drainage fluid. Only 12 (15%) of these isolates were from patients admitted to ICUs, and 6 of these 12 isolates were recovered in 1999. The incidence of VRE in enterococci causing nosocomial infections in ICUs was 7.0%. The relationship of increasing vancomycin use and the increase in vancomycin resistance in enterococci is shown in Figure 3.

**Figure 4:**
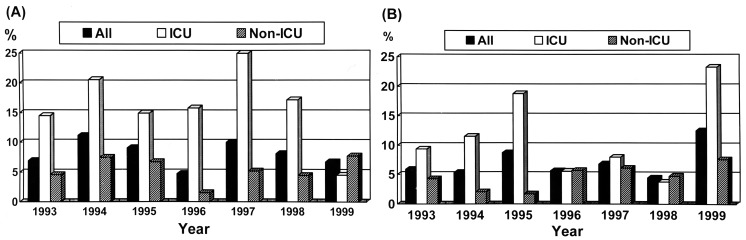
Proportions of *Pseudomonas aeruginosa* (A) and *Acinetobacter baumannii* (B) isolates causing nosocomial infections resistant to imipenem in National Taiwan University Hospital, 1993-1999. ICU = intensive care unit.

The incidences of imipenem-resistant *P. aeruginosa* (1993 to 1998) and imipenem-resistant *Acinetobacter baumannii* (1999) isolated from ICUs were five- to ten-fold higher than isolates recovered from non-ICU settings. However, this was not the case with imipenem-resistant *P. aeruginosa* in 1999 or imipenem-resistant *A. baumannii* in 1993-1998 (Figure 4).

All 150 isolates of *Candida* species were inhibited by 1 μg/mL of amphotericin B (MIC range 0.03 to 1 μg/mL). The MIC_50_ and MIC_90_ of *C. glabrata* were 16 μg/mL and >32 μg/mL, respectively. Twenty-one isolates (70%) of *C. glabrata* were nonsusceptible to fluconazole (MICs >8 μg/mL). Four (15.4%) of the *C. parapsilosis* isolates had MICs >8 μg/mL (2 had MICs 16 μg/mL and the other 2 had MICs 64 μg/mL).

## Discussion

Hospitals worldwide are continuing to face the crisis of the upsurge and dissemination of antimicrobial-resistant bacteria, particularly those causing nosocomial infections in ICU patients *1*,*27*–*29*. Among resistant bacteria, MRSA, MRCoNS, VRE, third-generation cephalosporin-resistant *Enterobacteriaceae*, and imipenem- or ciprofloxacin-resistant *P. aeruginosa* and *A. baumannii* are of great concern because these bacteria have spread worldwide and ultimately will compromise the antimicrobial therapy of infections caused by these organisms *2*,*25*–*28*,*30*.

This report describes trends in major nosocomial pathogens and shifts in antimicrobial resistance during a 19-year period in a large teaching hospital in Taiwan. In a comparison of data from a recent NNIS study and other surveillance systems *2*,*4*,*9*,*31*, our results suggest four conclusions. First, *Candida* species, rather than *P. aeruginosa*, *E. coli*, or staphylococci, now are the most frequent pathogens causing overall nosocomial infections and BSIs in this hospital. The upward trend in coagulase-negative staphylococci, which was the leading cause of BSI in the recent NNIS study, was not confirmed in our study. Second, we observed a remarkably high incidence of MRSA, especially in ICUs, contrasted with a relatively low incidence of VRE. Third, we report an increase in incidence of cefotaxime-resistant *Enterobacteriaceae*, especially in the incidence of cefotaxime-resistant *K. pneumoniae* in ICUs. Fourth, although the overall incidence of imipenem resistance among *P. aeruginosa* and *A. baumannii* in recent years has remained stable (4% to 11%), higher incidences of imipenem-resistant *P. aeruginosa* or *A. baumannii* were found in ICUs than in general wards in most time periods.

Since 1990, *Candida* species have continued to be an important cause of nosocomial BSI in the United States, and the proportion (40%-50%) of these infections due to species of *Candida* other than *C. albicans* may be increasing *(**12**,**19**,**20**,**31**,**32**)*. Among the species of *Candida* other than *C. albicans, C. glabrata* (prone to be resistant to fluconazole) and *C. krusei* (intrinsically resistant to fluconazole) are of clinical importance *31*,*33*. Although the proportion (40%) of candidemia due to non-*albicans* *Candida* species in 1999 in our hospital was similar to that (48%) reported in the United States from April 1995 to June 1996, the incidences of *C. glabrata* (8.8%) and *C. krusei* (0%) in our hospital were lower than those (20% and 5%, respectively) in the United States *31*.

Although an upsurge in the incidences of *K. pneumoniae* and *E. coli* isolates resistant to cefotaxime was noted in our ICUs, an investigation is under way into the mechanisms of resistance and potential outbreaks (clonal dissemination or gene transfer) *34*. The abrupt increase in the proportion of *A. baumannii* isolates resistant to imipenem in 1999 resulted from wide dissemination of several multidrug-resistant clones in ICUs and many general wards in the hospital (data not shown).

In summary, surveillance of the microbial etiology of nososcomial infections over prolonged time periods not only can provide important information for day-to-day decision making in antimicrobial therapy in individual hospitals but also can reflect local trends and shifts in etiology and antimicrobial drug resistance. Nosocomial pathogens have shifted away from easily treated bacteria toward more resistant bacteria and even to *Candida* species with fewer options for therapy. These shifts continue to present challenges for nosocomial infection control and prevention.
